# A Benchmark Dataset and Deep Learning-Based Image Reconstruction for Electrical Capacitance Tomography

**DOI:** 10.3390/s18113701

**Published:** 2018-10-31

**Authors:** Jin Zheng, Jinku Li, Yi Li, Lihui Peng

**Affiliations:** 1 Tsinghua National Laboratory for Information Science and Technology, Department of Automation, Tsinghua University, Beijing 100084, China; jennyzheng816@gmail.com (J.Z.); jinkuli123@163.com (J.L.); 2 Graduate School at Shenzhen, Tsinghua University, Shenzhen 518055, China; liyi@sz.tsinghua.edu.cn

**Keywords:** benchmark dataset, electrical capacitance tomography, machine learning, image reconstruction

## Abstract

Electrical Capacitance Tomography (ECT) image reconstruction has developed for decades and made great achievements, but there is still a need to find a new theoretical framework to make it better and faster. In recent years, machine learning theory has been introduced in the ECT area to solve the image reconstruction problem. However, there is still no public benchmark dataset in the ECT field for the training and testing of machine learning-based image reconstruction algorithms. On the other hand, a public benchmark dataset can provide a standard framework to evaluate and compare the results of different image reconstruction methods. In this paper, a benchmark dataset for ECT image reconstruction is presented. Like the great contribution of ImageNet that transformed machine learning research, this benchmark dataset is hoped to be helpful for society to investigate new image reconstruction algorithms since the relationship between permittivity distribution and capacitance can be better mapped. In addition, different machine learning-based image reconstruction algorithms can be trained and tested by the unified dataset, and the results can be evaluated and compared under the same standard, thus, making the ECT image reconstruction study more open and causing a breakthrough.

## 1. Introduction

Electrical capacitance tomography (ECT) is a measurement technique for visualizing dielectric multi-phase flow processes, such as pneumatic conveying systems and fluidized beds, by generating cross-sectional images [1,2,3]. A traditional ECT system mainly contains three parts: the ECT sensor, the capacitance measurement and data acquisition circuit, and the imaging computer. All the possible capacitance data among the non-redundant electrode combinations are measured based on a capacitance measurement circuit [4], and the permittivity distribution can be reconstructed by certain algorithms. For an ECT sensor with N electrodes, the number of available capacitance data is (N − 1) × N/2.

In the past three decades, research concerning ECT sensor design [5,6,7,8], hardware design [1,9,10,11,12], and image reconstruction algorithms [13,14,15,16,17,18,19,20,21] and applications [22,23,24,25,26,27,28,29,30] have been widely investigated and remarkable progress has been made. So far, ECT is still a very active field. The studies in the literature show that papers on the ECT field are published constantly, among which studies of ECT image reconstruction algorithms make up an important part of it.

Conventional algorithms such as the linear back project (LBP), the Landweber iteration, and the total variation (TV) based regularization are still adopted, meanwhile, in recent years, some distinctive works also have been reported. An example of such a distinctive work is the image reconstruction algorithm based on the sparsity constraint combined with the compressed sensing theory. Ye J. M. et al. designed an extended sensitivity matrix that consists of some normalized capacitance vectors corresponding to the base permittivity elements [31]. Zhao J. et al. used a sparse reconstruction by a separable approximation algorithm to solve the ECT inverse problem [32]. Yang Y. J. and Peng L. H. proposed an enhanced linear model and sparsity regularization for the image reconstruction algorithm [33]. In other areas, Taylor S. H. and Garimella S. V. adopted a level set method to reconstruct ECT images [34]. Ren S. J. et al. introduced the boundary element method for ECT image reconstruction, and this method was able to reconstruct the permittivity distribution profile in the imaging area well [35].

In recent years, machine learning theory has flourished in many fields and researchers in the ECT area have also attempted to introduce it to solve the image reconstruction problem. Marashdeh et al. trained a combined multilayer feed-forward neural network and analogue Hopfield network [36]. Wang et al. proposed a least square support vector machine and bacterial colony chemotaxis algorithm for ECT image reconstruction [37]. Li et al. attempted to make the BP and RBF neural networks solve the ECT image reconstruction problem [38]. Although these attempts made breakthroughs in ECT image reconstruction to some degree, most of these reported machine learning-based ECT image reconstruction methods are trained using a small-scale dataset that usually comprised of several tens to about one hundred instances. The generalization ability may be limited when the training dataset is small, which means that the training results may be good for the training dataset, but if given a new capacitance vector that the network has never seen before, the network may not be able to figure out the right corresponding permittivity distribution. So, a large-scale dataset is of great necessity for researchers in order to explore machine learning algorithms for ECT image reconstruction. However, there is still no public large-scale dataset in the ECT field.

As is known to all, a good public dataset, such as MNIST [39] and ImageNet [40] in the machine learning field, is a key part of machine learning research. For example, ImageNet, which is a large-scale dataset for researchers in the computer vision area, has millions of images under thousands of categories, with a typical category containing several hundred images [40]. Such a public dataset inspires researchers to explore faster and more accurate image classifying or object detecting methods and launches a great campaign to promote the development of machine learning, especially deep learning theory.

The ImageNet example shows that not only models should be emphasized, but data should also be treated with more attention. The availability of more data would help researchers develop better algorithms. A free and open large-scale dataset is also expected (in the ECT field) to contribute more data to better map the relationship between capacitance and the permittivity distribution and to evaluate and compare the results of different image reconstruction methods under the same criteria.

On the other hand, in order to get the required amount of ECT capacitance and permittivity distribution data for a study on image reconstruction, a lot of simulation models need to be built or a practical ECT experiment system needs to be established, which will cost much in both the materials and time. However, when a large-scale benchmark dataset is brought out, researchers will find it convenient since they need not repeat their data acquisition work. Like the great benefits from ImageNet, it is hoped that such a large-scale public benchmark ECT dataset would also make researchers realize the importance of the dataset, start a revolution of solving the image reconstruction problem, encourage researchers to explore better image reconstruction methods, and have more communication, leading to a breakthrough in ECT image reconstruction theory.

In this paper, a benchmark dataset for ECT image reconstruction is proposed. It consists of tens of thousands of capacitance vectors and corresponding permittivity distribution vectors, as well as sensitivity matrices obtained from 2D simulation models, and 3D simulation models along with static and dynamic experiments. The benchmark dataset can be regarded as two parts. One subset, whose data is from the 2D simulation models, is for the training and testing of machine learning methods for ECT image reconstruction. The other subset, whose data is from 3D simulation models and experiments, is for evaluating and comparing different ECT image reconstruction algorithms. This study is concerned with typical two-phase flow patterns—annular, stratified, single bar, and two-bar. Additionally, three relative permittivity values—2.7, 3.8, and 80—are set on the phase in the higher permittivity value and the lower permittivity value is set to 1. The image reconstruction results of the three traditional algorithms, i.e., the LBP, the projected Landweber iteration, and the total variation (TV) based regularization, along with the deep learning-based method proposed in Reference [41] are used as examples on how to compare different algorithms under the same evaluation criteria of the benchmark dataset.

The paper is organized as follows. Section 2 and Section 3 provide the benchmark dataset and the image reconstruction result examples based on the simulations and experiments, respectively. Finally, conclusions are drawn in Section 4.

## 2. The Simulation Part of the Benchmark Dataset

In this section, the simulation part of the benchmark dataset based on 2D and 3D models is introduced. Permittivity distribution images are reconstructed by capacitance vectors in the dataset based on 3D models by using the three conventional ECT image reconstruction algorithms—i.e., the LBP, the projected Landweber iteration, and the TV-based regularization—as well as by using a machine learning-based image reconstruction method. The quantitative criteria for the comparison of the image reconstruction results are also provided.

### 2.1. The Simulation Part of the Benchmark Dataset Based on the 2D Models

One important part of the benchmark dataset is a large-scale dataset built as a public database for the training and testing of the machine learning-based ECT image reconstruction algorithms. The large-scale dataset is generated by a platform which is established on MATLAB with a GUI and worked on with 2D 8-electrode ECT sensor models built on the finite element analysis software, COMSOL Multiphysics [42]. It contains totally 40,000 pairs of ECT data samples, with each pair of samples consisting of a normalized permittivity distribution vector with 3228 elements and the corresponding normalized capacitance vector of with an 8-electrode ECT sensor with 28 elements. The flow patterns of the samples are annular, stratified, single bar, and two-bar, respectively. Additionally, each flow pattern has 10,000 pairs of samples.

**Figure 1:**
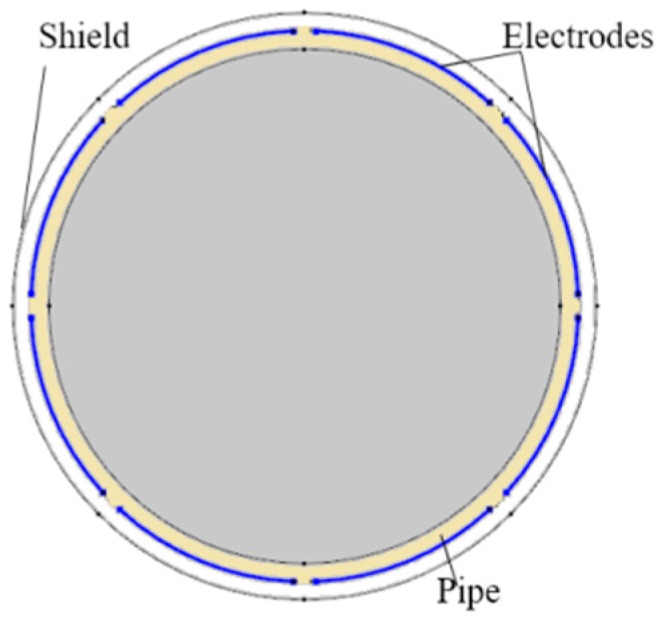
The structure of the 2D simulation model.

The 8-electrode ECT sensor model in COMSOL Multiphysics is shown in Figure 1. The material of the sensor pipe is set to be PVC with a relative permittivity of 2. The lower and higher permittivity values of the flow are 1 and 2.7, respectively. The diameter of the pipe is 70 mm and the thickness of the pipe is 3.5 mm. The gap between two adjacent electrodes is 5 degrees so that the span angle of each electrode is 40 degrees. The round imaging cross-section is divided into a 64 × 64 mesh grid which, in total, has 3228 effective pixels.

**Figure 2:**
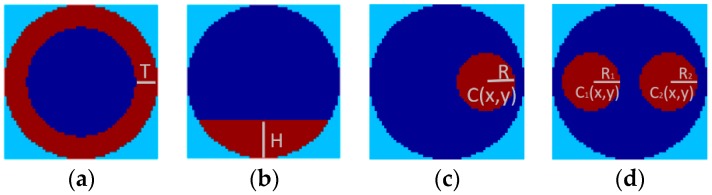
The four flow patterns in the dataset. (**a**) annular; (**b**) stratified; (**c**) single bar; (**d**) two-bar.

The four flow patterns chosen for the benchmark dataset are typical two-phase flow patterns that commonly occur in the industrial field, and other complex flow patterns can be regarded as combinations of these flows. Although the names of these four flow patterns may not be the same—for example, the single bar flow is also called the core flow and two-bar flow may be mentioned as the two-object flow—they are mostly studied in ECT image reconstruction research, such as those reported in References [1,12,14,16]. To describe the phantoms of different flow patterns quantitatively, certain parameters are selected. The parameter describing annular flow is the thickness of the annular, which is normalized with respect to the radius of the sensor and denoted by T. For the stratified flow, the normalized height of the flow surface is selected, i.e., H. For the single bar, the position of the center point C(x,y) of the bar, of which the coordinates are normalized with respect to the sensor radius, is also used besides the normalized bar radius, R. For the two-bar distribution, the normalized radii—i.e., R1 and R2—and the positions of the center points of the two bars, i.e., C_1_(x,y) and C_2_(x,y), are all used. Figure 2 depicts the four flow patterns with the corresponding parameters.

### 2.2. The Simulation Part of the Benchmark Dataset Based on the 3D Models

Another simulation part of the benchmark dataset for evaluating and comparing the ECT image reconstruction algorithms is also built based on 3D models. This part contains capacitance vectors corresponding to 80 cases, including the capacitance vectors of the full and empty pipes for calibration, 2 sensitivity matrices for the 8-electrode sensor, and the 12-electrode sensor, respectively, and 12 normalized permittivity distribution vectors.

The four flow patterns and the pixel division of the samples in the dataset based on the 3D simulation models are the same as those in Section 2.1. Three relative permittivity values—2.7 (e.g., oil), 3.8 (e.g., sand), and 80 (e.g., water)—are investigated, covering situations of low-contrast and high-contrast permittivity changes. Table 1 provides normalized parameters describing the phantoms and the corresponding phase ratio of the material with a high permittivity in each phantom.

**Figure 3:**
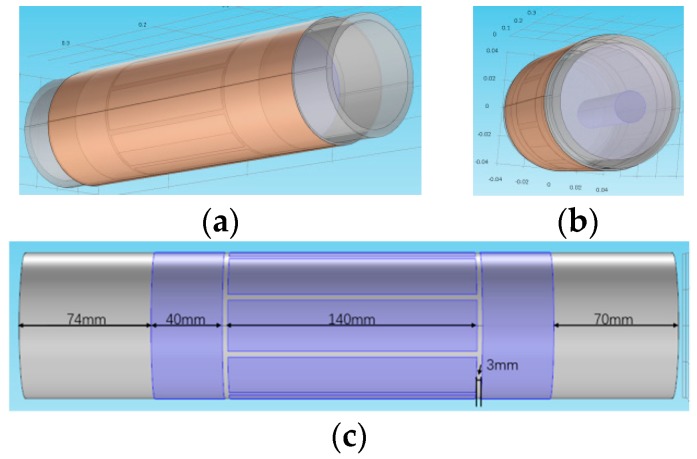
The 3D simulation model of the 8-electrode ECT sensor. (**a**) A 3D view of the sensor; (**b**) a 3D view of a single bar flow; (**c**) the length of each part of the sensor.

To obtain the simulated capacitance data regarding the different phantoms, a 3D 8-electrode ECT sensor model and a 3D 12-electrode ECT sensor model are built in the COMSOL Multiphysics software. Figure 3 depicts the 3D 8-electrode ECT sensor model for the simulation. The inner diameter of the pipe is 70 mm and the outer diameter is 80 mm. The length of the sensor is 370 mm, of which the electrode length is 140 mm. The gap between the two adjacent electrodes is 5 degrees so that the span angle of each electrode is 40 degrees and 25 degrees for the 8-electrode sensor and the 12-electrode sensor, respectively.

Considering both the computed accuracy and time cost, the capacitance data in this benchmark dataset are computed based on a custom mesh in COMSOL Multiphysics with the maximum element size set to 20.4 mm, the minimum element size set to 1.48 mm, and the maximum element growth rate set to 1.4. For the empty pipe case, the total mesh element number is 1,119,690.

**Figure 4:**
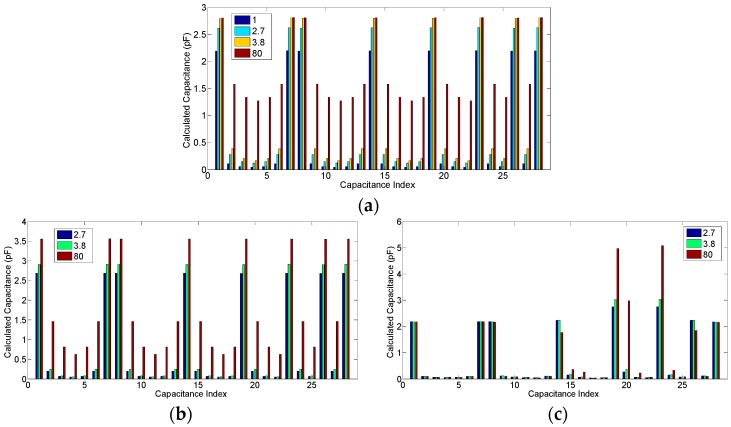

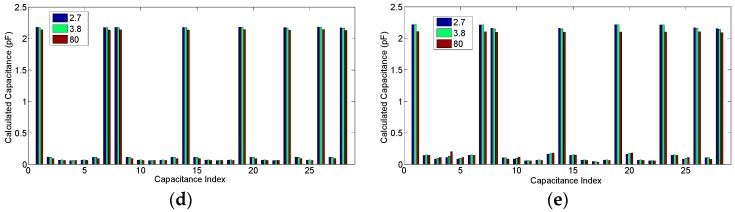
The capacitance data examples based on the simulation, (**a**) the empty pipe and full pipe; (**b**) the 50% annular distribution; (**c**) the 19.58% stratified distribution; (**d**) the 13.88% single bar distribution; (**e**) the 27.76% two-bar distribution.

The capacitances among the different electrode combinations are dependent on the relative permittivity, the phase ratio, and the flow pattern. Figure 4 is an example of how these factors matter, where the capacitance vectors of each flow pattern with one phase ratio under three different permittivity values are compared, and the capacitance vectors of the empty pipe and the full pipe under these three permittivity values are also given for calibration. All these capacitance values are simulated based on the 8-electrode sensor. The corresponding capacitance data are given in Table 2.

For an infinite parallel-plate capacitor, the capacitance value increases along with an increase of the permittivity value of the medium between the two electrode plates so that the relationship between variation of the capacitance and the variation of the permittivity value is linear. However, it can be found that for ECT sensor, the relationship between the capacitance value and the permittivity value is nonlinear, especially for the case in which the permittivity variation is of a high contrast. In Figure 4a, the capacitance values of the adjacent electrode pairs with permittivity values of 3.8 and 80 are very close and, in Figure 4d,e, the capacitance values of the adjacent electrode pairs with a permittivity value of 80 are even smaller than those with permittivity values of 2.7 and 3.8.

**Figure 5:**
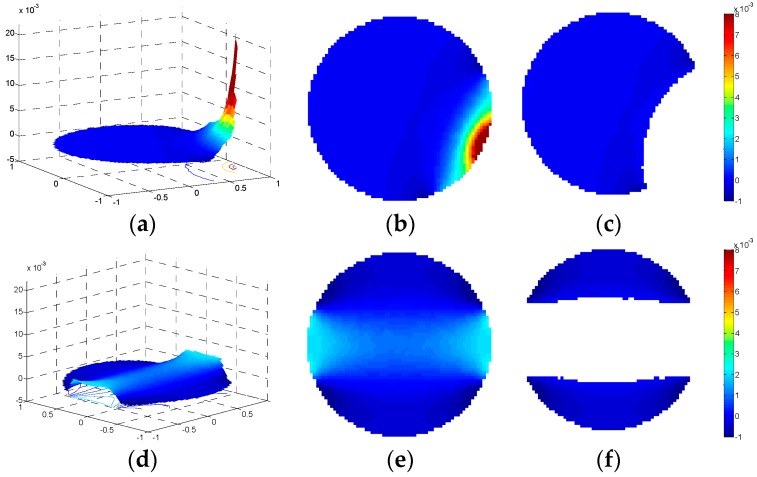
The sensitivity map of the 8-electrode ECT sensor, (**a**) a 3D view, adjacent electrode pair; (**b**) a 2D view, adjacent electrode pair; (**c**) a 2D view of the negative sensitivity zone, adjacent electrode pair; (**d**) a 3D view, opposite electrode pair; (**e**) a 2D view, opposite electrode pair; (**f**) a 2D view of the negative sensitivity zone, opposite electrode pair.

This phenomenon appears because, for adjacent electrode pairs, only a very small region in the circular ECT imaging area has a very sharp positive sensitivity while most of the region has a negative sensitivity. Meanwhile, for the opposite electrode pairs, most of the region in the circular ECT imaging area has a relatively high positive sensitivity while a relatively small region has a negative sensitivity. The comparisons of the sensitivity map appearance of the adjacent electrode pairs and the opposite electrode pairs regarding the negative sensitivity characterizations are demonstrated clearly in Figure 5. The effect of the negative sensitivity map can also be reflected from the capacitance values of the adjacent electrode pairs while the permittivity value is 80 and the distributions are annular or stratified. It was found from Figure 4b that the capacitance values of the adjacent electrode pairs, while the permittivity distribution is 50% annular, are about 3.55 pF, which are even larger than the values while the pipe is full. Furthermore, for the 19.58% stratified distribution (Figure 4c), certain capacitance values of the adjacent electrode pairs reach 5.08 pF.

With the change of the medium’s permittivity from a low value to a high value in the ECT imaging area, the capacitance values among the different electrode pairs behave totally differently in terms of their properties and nonlinearities. Cui et al. [43] and Yang et al. [44] reported and preliminarily analyzed the effect of the nonlinearity of capacitances between different electrode pairs on ECT image reconstruction. This issue may need to be investigated more deeply in future studies on ECT image reconstruction, particularly while the permittivity distribution inside the sensor has a relatively high contrast variation.

### 2.3. The Deep Autoencoder and the Iteration Method Based on It

As is known in the ECT field, the nonlinear relationship between capacitance and permittivity deteriorates the quality of the reconstructed image based on the linear model when the permittivity variation becomes large. This is because the linear model approximates the nonlinear relationship between capacitance data and the corresponding permittivity distribution by neglecting the higher order terms of permittivity variation. When the permittivity variation becomes larger, the neglected terms matters more, thus, the imaging reconstruction quality worsens. However, if the nonlinear model is used for improving the quality of the image reconstruction, the real-time ability of the nonlinear model-based algorithms for online imaging should be considered. In this sense, better image reconstruction algorithms should be put forward to meet the requirements of both imaging quality and speed.

**Figure 6:**
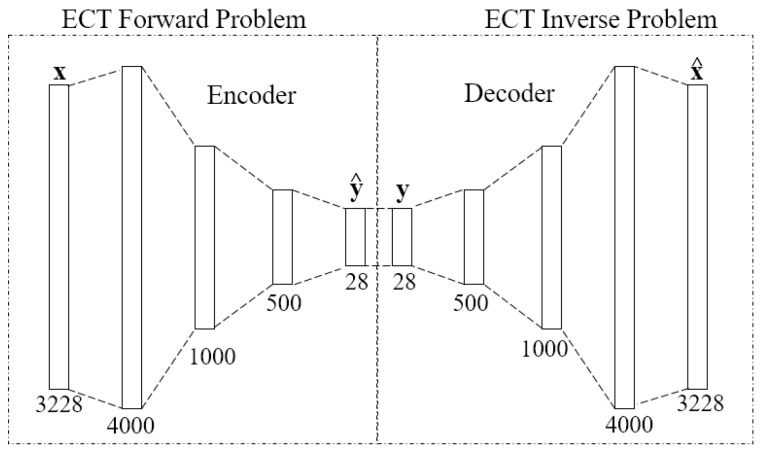
The structure of the deep autoencoder.

A deep autoencoder along with the iteration method proposed in Reference [41] provides a new way to solve the ECT image reconstruction problem. This method is a deep supervised autoencoder which has an encoder and a decoder (with five layers each) that can deal with both the former problem and the inverse ECT problem. The nonlinear relationship from the permittivity distribution to the capacitance data is modeled by the encoder (*F*(·)) and, conversely, the reconstruction from the capacitance data to the permittivity distribution is solved by the decoder (*G*(·)) of the deep autoencoder. Suppose **x** is the vector of the permittivity distribution, **y** is the capacitance data vector, $\hat{x}$ is the reconstructed permittivity distribution, and $\hat{y}$ is the estimated capacitance data calculated from the permittivity distribution, then, according to the structure of the autoencoder in Figure 6, there is

(1) $$\left\{ \begin{array}{l} \hat{y}=F(x)\\ \hat{x}=G(y) \end{array}\right.$$

To take into account both the forward problem and the inverse ECT problem under the deep autoencoder framework, another two vectors—$\tilde{x}$ and $\tilde{y}$—are defined as follows:

(2) $$\left\{ \begin{array}{l} \tilde{y}=F(\hat{x})=F(G(y))\\ \tilde{x}=G(\hat{y})=G(F(x)) \end{array}\right.$$

The autoencoder is trained by minimizing the loss function, which is denoted by *L*. Because of the four estimated variables in Equations (1) and (2), *L* consists of four parts, see Equation (3), where $\alpha_{1}$, $\alpha_{2}$, $\alpha_{3}$, and $\alpha_{4}$ are the weights of these four parts of losses, *l* is a particular reconstruction error which chosen to be mean squared error (MSE), as is described in Equation (4), for any two *n*-dimensional vectors **v** and $\hat{v}$.

(3) $$\begin{array}{ll} L & =\alpha_{1}L_{1}+\alpha_{2}L_{2}+\alpha_{3}L_{3}+\alpha_{4}L_{4}\\ & =\alpha_{1}l(y,\hat{y})+\alpha_{2}l(x,\hat{x})+\alpha_{3}l(y,\tilde{y})+\alpha_{4}l(x,\tilde{x}) \end{array}$$

(4) $$l(\;v,\hat{v})=\frac{1}{n}\sum_{i=1}^{n}{\left(v_{i}-{\hat{v}}_{i}\right)}^{2}$$

Although the proposed deep autoencoder would take a lot of time to train, when it is well-trained, the deep autoencoder can be faster than most traditional ECT image reconstruction algorithms where the forward problem is usually solved by some time-consuming finite element method (FEM) and the image reconstruction algorithm would also consume a lot of calculation resources. Some iterative algorithms even need to repeatedly solve the forward problem and the inverse problem, which will lead to a good image reconstruction quality but will sacrifice too much time to satisfy its online use. If the deep autoencoder is used to implement the iterative process, the calculation time can be saved and image reconstruction quality will be promoted. Thus, an iteration method is inspired by the following Landweber iteration [45]:

(5) $${\hat{x}}_{k+1}={\hat{x}}_{k}-\alpha_{k}S^{T}(S{\hat{x}}_{k}-y)$$

where **x***_k_* is the calculated permittivity distribution at the *k*th step and **y** is the normalized capacitance vector. **S** is the sensitivity map in the linear model of the ECT, which maps the permittivity distribution to the capacitance data and corresponds to *F*(·) in Equation (1), and **S***^T^* maps the capacitance data to the permittivity distribution as *G*(·). So, if the deep autoencoder is used to implement the Landweber iteration, the equation should be Equation (6).

(6) $${\hat{x}}_{k+1}={\hat{x}}_{k}-\alpha_{k}G(F({\hat{x}}_{k})-y)$$

### 2.4. Image Reconstruction Examples Based on the Simulation

In this paper, four image reconstruction algorithms, i.e., the LBP [46], the projected Landweber [45], the total variation (TV) based regularization algorithm [47], and the deep autoencoder introduced above, are executed on the 3D model-based simulation part of the benchmark dataset. In order to quantitatively evaluate the ECT image reconstruction results and compare the performance of the different reconstruction algorithms, the evaluation criteria should be determined. The commonly used criteria include the relative image error of the reconstruction, the correlation coefficient between the real permittivity distribution and reconstructed permittivity distribution, and the other parameters related to the permittivity distribution, such as the phase ratio (phase concentration).

1. Relative image error

The relative image error is defined as the relative error of the reconstructed permittivity vector $\hat{g}$ with respect to the real permittivity vector g, as is shown below:

(7) $$\mathrm{Relative}\;\mathrm{image}\;\mathrm{error}=\frac{\left\|\hat{g}-g\right\|}{\left\|g\right\|}$$

2. Correlation coefficient

The correlation coefficient indicates the similarity between the reconstructed permittivity distribution and the original permittivity distribution, which is defined as Equation (8), where $\bar{\hat{g}}$ is the mean of $\hat{g}$, ${\hat{g}}_{i}$ is the *i*th element of $\hat{g}$, $\bar{g}$ is the mean of g, and $g_{i}$ is the *i*th element of g.

(8) $$\mathrm{Correlation}\;\mathrm{coefficient}=\frac{\sum_{i=1}^{N}\left({\hat{g}}_{i}-\bar{\hat{g}})(g_{i}-\bar{g}\right)}{\sqrt{\sum_{i=1}^{N}{\left({\hat{g}}_{i}-\bar{\hat{g}}\right)}^{2}\sum_{i=1}^{N}{\left(g_{i}-\bar{g}\right)}^{2}}}$$

3. Phase ratio error

The ECT image reconstruction is commonly used for evaluating the phase ratio in the application of the two-phase flow measurement, thus, the phase ratio error of the reconstructed image is also an important criterion. In this paper, the ‘phase ratio’ is the phase concentration of the medium with the higher permittivity value, which is computed by summing the permittivity distribution vector, i.e., the gray-scale value of the permittivity distribution. By using $R_{r}$ to stand for the real phase ratio, which can be calculated according to the phantom, and $R_{e}$ to stand for the estimated phase ratio from the reconstructed permittivity distribution, the phase ratio error can be defined as

(9) $$\mathrm{Phase}\;\mathrm{ratio}\;\mathrm{error}=R_{e}-R_{r}$$

**Figure 7:**
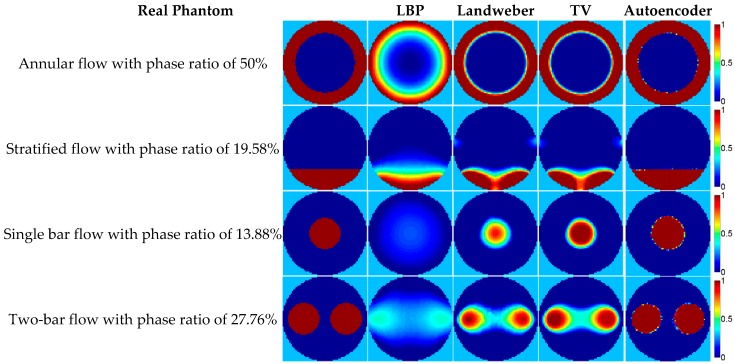
The image reconstruction examples based on the 3D simulation.

Some image reconstruction examples calculated by the 8-electrode capacitance vectors in the four flow patterns with a relative permittivity of 2.7 in Table 2 are shown in Figure 7, where the comparison of the image reconstruction results by different reconstruction algorithms is demonstrated. The related criteria data are listed in Table 3. Note that the phase ratio is estimated by summing the reconstructed normalized permittivity vector, and there is an artifact in the reconstructed images, therefore, the phase ratio error does not have a positive correlation with the relative image error.

In Figure 7, the images reconstructed by the autoencoder are apparently much better than that the images constructed by the other three traditional algorithms in terms of the visual effect, and very close to their corresponding real permittivity distributions. As for the other three algorithms, the reconstructed images of the LBP are far from the real permittivity distributions, especially for the single bar and two-bar flow. This conclusion can also be supported by the results of the three criteria in Table 3: all the criteria data show that the quality of image reconstruction by the autoencoder is much better than those constructed by the three traditional algorithms. The image reconstruction quality of the LBP is worse than the projected Landweber iteration and the TV. As for the reconstruction results of the projected Landweber iteration and the TV, they are much more similar to the real permittivity distribution than the LBP results visually, however, when compared to the criteria data in Table 3, it can be found that the projected Landweber iteration and the TV perform similarly in the two annular flows and two stratified flows cases, but the TV algorithm shows a better performance in the single bar and two-bar flows cases evaluated by all the criteria data.

**Figure 8:**
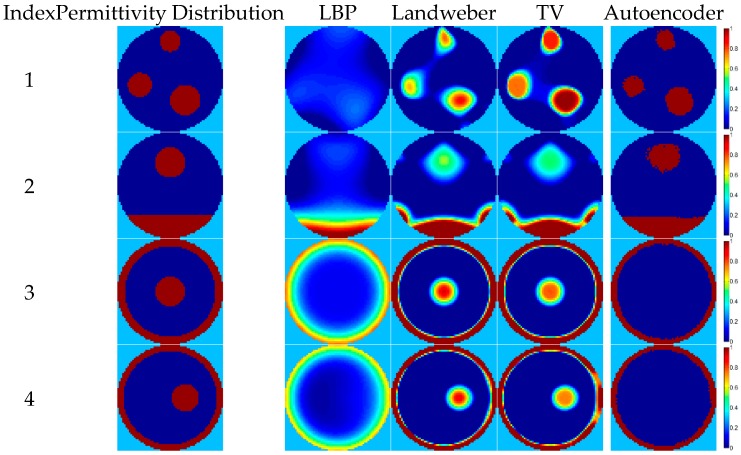
The image reconstruction examples of flow patterns not in the training dataset.

Figure 7 also shows that although the autoencoder is trained by the 2D simulation dataset, its performance is still satisfying in the 3D simulation dataset. This means that the autoencoder has a generalization ability to some extent. In order to further examine the generalization ability of the autoencoder, some flow patterns not in the training dataset are tested; see Figure 8.

**Figure 9:**
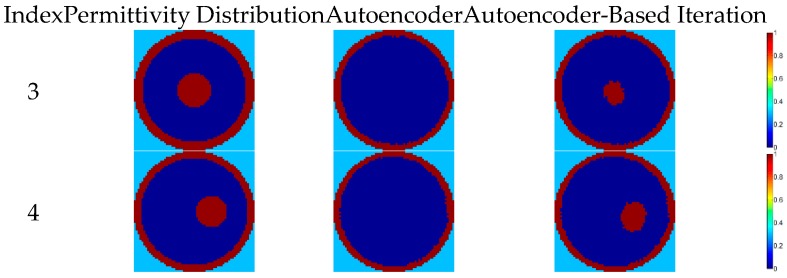
The image reconstruction examples of the iteration method.

Because there are only four flow patterns in the training dataset (i.e., annular, stratified, single bar, and two-bar), the performance of recognizing other new flow patterns with the proposed autoencoder network depends on its generalization ability. The results of the autoencoder are better than the other three algorithms in phantom 1 and 2 of Figure 8, showing that the autoencoder does have some generalization ability. However, the results of phantom 3 and 4 are quite unsatisfactory and bars inside the annulus cannot be reconstructed completely, implying that the generalization ability of the autoencoder is not good enough to recognize all of the new flow patterns. In order to promote the image reconstruction quality of the deep autoencoder, the iteration method introduced in Section 2.3 is implemented; see Figure 9. After using the iteration method, the image reconstruction results are improved as shown in Figure 9 and bars inside the annulus can be recognized because there is a single bar flow in the training dataset. A good way of improving the generalization ability is by enhancing the diversity of the training data, so, in future, more other flow pattern data are considered to be supplemented in the dataset to increase the generation ability of the methods based on machine learning.

## 3. The Experiment Part of the Benchmark Dataset

The experiment part of the benchmark dataset is also built. This includes the static experiment data of the 8-electrode capacitance vectors of the four flow patterns, each under the three situations, and empty and full pipes for the calibration. Besides, three capacitance vectors without other information are given to researchers who are interested in ECT image reconstruction in order to test their algorithms. Dynamic experiment data are also included in the dataset. The experiment devices and image reconstruction examples based on the experimental data are given in this section.

### 3.1. The Static Experiment Part of the Benchmark Dataset

**Figure 10:**
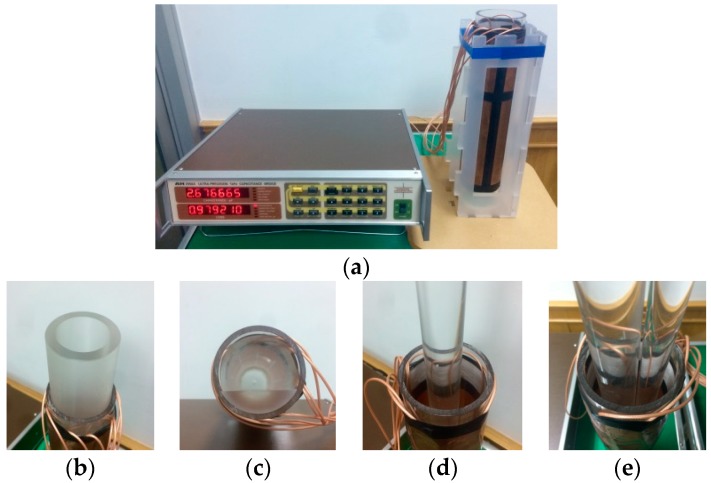
The static experiment setup, (**a**) the capacitance bridge and ECT sensor; (**b**) the annular distribution; (**c**) the stratified distribution; (**d**) the single bar distribution; (**e**) the two-bar distribution.

The Andeen–Hagerling high-precision capacitance bridge (AH-2550A) is used to measure the capacitance. Figure 10a shows the static experiment scenario, where the machine to the left is an AH capacitance bridge and that to the right is an 8-electrode ECT sensor with a support. Figure 10b–e show how the four flow patterns are implemented in the static experiment.

The ECT sensor is in the same structure as the 3D simulation model in Section 2.2. The pipe is made by acrylic (PMMA), the relative permittivity of which is considered to be near 3.8. The media that construct the flow patterns are also acrylic. The flow patterns are the same four types that are in the simulation: annular, stratified, single bar, and two-bar. Each of the flow patterns concludes 3 cases in terms of the corresponding parameter and phase ratio; see Table 4.

**Figure 11:**
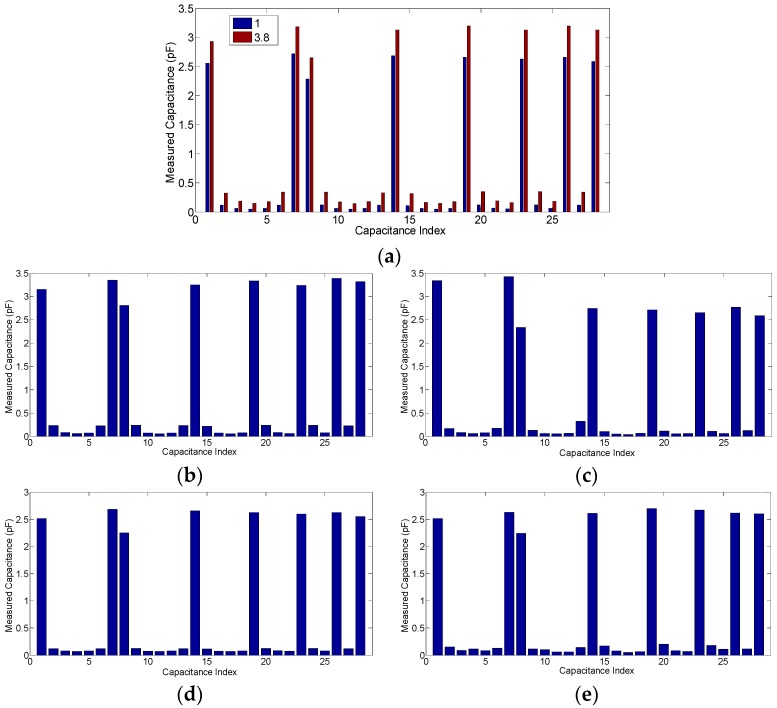
The static experiment capacitance data examples while the permittivity value is 3.8, (**a**) the empty pipe and full pipe; (**b**) the 48.95% annular distribution; (**c**) the 19.58% stratified distribution; (**d**) the 18.31% single bar distribution; (**e**) the 26.52% two-bar distribution.

Figure 11 shows the capacitance data chosen from one case of each phantom, the phase ratio of which is 48.95% in the annular flow (the annular thickness is 10 mm), 19.58% in the stratified flow (the stratified height is 17.5 mm), 18.31% in the single bar (the bar radius is 15 mm), and 26.52% in the two-bar (the two bar radii are 10 mm and 15 mm, respectively). The corresponding capacitance data are listed in Table 5.

It was found that the capacitance values in Figure 11a are slightly different from those corresponding to the simulation-based values in Figure 4a. In addition, even the capacitance data with similar geometric relationships, such as the capacitance values of the adjacent electrode pairs, are slightly different from each other. The reason for this is that the ECT sensor used in the experiment, due to manufacturing precision limitations, is not absolutely geometrical symmetrical and identical to the sensor model used in the simulations. However, from the image reconstruction point of view, these differences do not affect the image reconstruction results too much because only the normalized capacitance data are used.

### 3.2. The Image Reconstruction Examples Based on the Static Experiment

**Figure 12:**
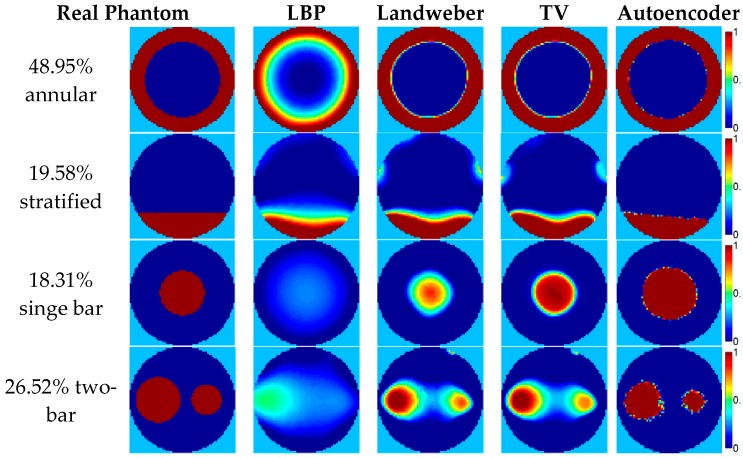
The image reconstruction examples based on the static experiment data.

The four ECT image reconstruction algorithms used in Section 2.3 are also executed using the static experiment capacitance data. The sensitivity matrix used for the three traditional algorithms is that which was generated in the 3D simulation. The image reconstruction results based on the capacitance vectors in Table 5 are shown in Figure 12. The comparison of the image reconstruction results in Figure 12 are listed in Table 6.

### 3.3. The Capacitance Data Open for the Image Reconstruction Study

Three measured capacitance vectors, the permittivity distribution information of which are not open to the public, are given in Table 7. The empty and full pipes’ capacitance vectors for calibration can be found in Table 5. These three capacitance vectors are published for researchers who are interested in ECT image reconstruction in order to estimate what the real phantoms are and to evaluate their own algorithms. In terms of the sensitivity matrix, researchers can use their own calculated matrices based on the 3D ECT sensor, as described in this paper, or they can ask for the one used in this paper by email.

### 3.4. The Dynamic Experiment Part of the Benchmark Dataset

**Figure 13:**
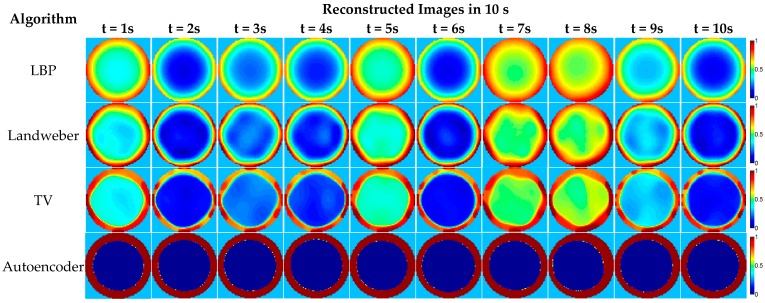
The image reconstruction examples based on the dynamic experiment data.

The dynamic experiment part of the dataset is the capacitance values of the oil-gas two-phase flow given in the form of normalized capacitance data sequences, which are obtained from an experimental test rig with a pipeline with a 50 mm diameter. The testing ECT system has an 8-electrode sensor and is installed on a vertical Venturi throat section. Before flowing through the Venturi pipe, the oil and gas are separately issued and then mix as a two-phase flow. The data acquisition software in the upper computer records capacitance data are measured using the ECT sensor and transformed using the data acquisition circuit. The measurement system is calibrated by using a pipe full of oil and a pipe full of air. The oil-gas two-phase flows with the different gas volume fractions (GVFs) are measured. The dataset includes three samples whose GVF and corresponding flow rates are given in Table 8. The normalized capacitance data sequence of 62.09% GVF is given in Table 9 as an example and the corresponding reconstructed images are given in Figure 13.

## 4. Conclusions

In this paper, a benchmark dataset for ECT based on 2D and 3D simulations, as well as static and dynamic experiments, is built. The 2D simulation part contains 40,000 pairs of samples with normalized capacitance vectors and their corresponding permittivity distribution vectors. The 3D simulation part contains capacitance vectors corresponding to 80 cases, including capacitance vectors of full and empty pipes for calibration, 2 sensitivity matrices for the 8-electrode model and the 12-electrode model, respectively, as well as 12 normalized permittivity distribution vectors. The static experiment part contains 14 capacitance vectors of the 14 cases, along with 3 capacitance vectors without flow pattern information. The dynamic experiment part contains three normalized capacitance data sequences in different GVFs.

Among these four parts of the benchmark dataset, the part based on the 2D simulation is used as the public database for researchers to use in training and testing their own machine learning-based ECT image reconstruction algorithms. Additionally, the other three parts of the benchmark dataset—i.e., the 3D simulation part, the static experiment part, and the dynamic experiment part—can be used as a benchmark for evaluating and comparing the different ECT image reconstruction methods. Three criteria—i.e., the relative image error, the correlation coefficient, and the ratio error—are put forward as the quantitative standard to evaluate and compare the ECT image reconstruction methods. The LBP, the projected Landweber, the total variation (TV) based regularization algorithm, and a deep learning method based on an autoencoder for ECT are used as examples of how to compare the different algorithms under the same evaluation criteria of our benchmark dataset. They are executed in the 3D simulation part, the static experiment part, and the dynamic experiment part of the benchmark dataset, respectively, and the corresponding image reconstruction results are evaluated using the three criteria. Most visual results and quantitative results show that the autoencoder-based deep learning method can perform better reconstructions than the three traditional algorithms and that it has a good generalization ability. However, some results show that the autoencoder is not perfect and that the generalization ability can be further improved.

The benchmark dataset that supported the new deep learning-based image reconstruction algorithm is still at its initial stage and it is not perfect enough at present. It mainly focuses on the research of data from mostly used the 8-electrode and 12-electrode ECT sensors, and there are only four types of flow patterns in the benchmark dataset. Supplements to the benchmark dataset could enhance the diversity of the training dataset for machine learning-based image reconstruction methods, improve the performance of these methods, and expand their application range. In the future, we will add more simulation and experiment data to improve this benchmark dataset. We also welcome other researchers to contribute to the dataset by integrating data from other ECT sensor models—including the 16-electrode ECT sensor, the 3D ECT sensor, and dates related to other flow patterns—and to evaluate their new image reconstruction algorithms under the criteria of the benchmark dataset.

We hope this benchmark dataset can be used by researchers to try new image reconstruction methods—especially faster and better methods based on machine learning, where the hardware system or the simulation model is not necessary—and to make the ECT image reconstruction research area more open and flexible, leading to a big breakthrough.

## Figures and Tables

**Table 1:** The phantom parameters of the 3D simulation part of the benchmark dataset.

Flow Pattern	Parameter				Phase Ratio
Annular	**T**				
	0.05				10%
	0.30				50%
	0.55				80%
Stratified	**H**				
	0.25				19.58%
	0.50				50%
	0.75				80.42%
Single Bar	**C(x,y)**		**R**		
	(0,0)		0.29		7.93%
	(0,0)		0.37		13.88%
	(0.5,0)		0.50		25%
Two-bar	**C1(x,y)**	**C2(x,y)**	**R1**	**R2**	
			0.29	0.29	15.86%
	(−0.5,0)	(0.5,0)	0.29	0.37	21.81%
			0.37	0.37	27.76%

**Table 2:** The capacitance data related to the examples in Figure 4 (in pF).

Electrode Pair	Empty and Full Pipe				50% Annular			19.58% Stratified			13.88% Single Bar			27.76% Two-Bar		
*ε*	1	2.7	3.8	80	2.7	3.8	80	2.7	3.8	80	2.7	3.8	80	2.7	3.8	80
**1-2**	2.191	2.615	2.796	2.807	2.681	2.907	3.552	2.182	2.178	2.176	2.179	2.173	2.224	2.218	2.220	2.107
**1-3**	0.110	0.282	0.382	1.579	0.195	0.246	1.463	0.103	0.101	0.096	0.112	0.112	0.151	0.142	0.151	0.144
**1-4**	0.056	0.147	0.203	1.337	0.069	0.077	0.806	0.062	0.064	0.061	0.068	0.071	0.082	0.085	0.095	0.106
**1-5**	0.046	0.122	0.170	1.273	0.052	0.055	0.627	0.060	0.063	0.063	0.062	0.066	0.069	0.106	0.130	0.200
**1-6**	0.056	0.147	0.203	1.338	0.069	0.077	0.807	0.062	0.064	0.061	0.068	0.071	0.082	0.085	0.095	0.106
**1-7**	0.110	0.283	0.383	1.579	0.195	0.247	1.463	0.103	0.101	0.097	0.112	0.112	0.151	0.142	0.151	0.144
**1-8**	2.199	2.623	2.804	2.812	2.686	2.912	3.556	2.187	2.187	2.184	2.174	2.169	2.223	2.213	2.215	2.102
**2-3**	2.193	2.617	2.799	2.808	2.681	2.907	3.552	2.182	2.179	2.169	2.179	2.173	2.169	2.159	2.149	2.098
**2-4**	0.110	0.282	0.382	1.579	0.195	0.246	1.462	0.122	0.124	0.097	0.112	0.112	0.107	0.105	0.103	0.084
**2-5**	0.056	0.147	0.203	1.338	0.069	0.077	0.807	0.073	0.077	0.081	0.068	0.071	0.057	0.087	0.097	0.110
**2-6**	0.046	0.122	0.170	1.274	0.052	0.055	0.627	0.052	0.054	0.068	0.062	0.066	0.052	0.058	0.060	0.056
**2-7**	0.056	0.147	0.204	1.339	0.069	0.077	0.807	0.048	0.047	0.044	0.068	0.071	0.071	0.067	0.070	0.064
**2-8**	0.110	0.283	0.383	1.580	0.195	0.246	1.462	0.106	0.105	0.102	0.112	0.112	0.167	0.161	0.175	0.182
**3-4**	2.196	2.620	2.801	2.810	2.681	2.908	3.552	2.231	2.228	1.765	2.177	2.171	2.186	2.159	2.149	2.096
**3-5**	0.110	0.283	0.383	1.579	0.195	0.246	1.462	0.158	0.177	0.366	0.112	0.112	0.104	0.144	0.153	0.148
**3-6**	0.056	0.147	0.203	1.338	0.069	0.077	0.807	0.065	0.071	0.258	0.068	0.071	0.051	0.067	0.069	0.063
**3-7**	0.046	0.122	0.170	1.274	0.052	0.055	0.628	0.036	0.034	0.041	0.062	0.066	0.045	0.045	0.044	0.034
**3-8**	0.056	0.147	0.203	1.338	0.069	0.077	0.807	0.048	0.047	0.044	0.068	0.071	0.071	0.067	0.069	0.063
**4-5**	2.196	2.620	2.801	2.809	2.680	2.906	3.550	2.742	3.016	4.974	2.182	2.176	2.190	2.217	2.218	2.099
**4-6**	0.110	0.283	0.383	1.579	0.195	0.246	1.462	0.268	0.370	2.984	0.112	0.112	0.105	0.161	0.175	0.182
**4-7**	0.056	0.147	0.204	1.338	0.069	0.077	0.807	0.065	0.070	0.227	0.068	0.071	0.051	0.067	0.070	0.064
**4-8**	0.046	0.122	0.170	1.274	0.052	0.055	0.627	0.052	0.053	0.066	0.062	0.066	0.052	0.058	0.060	0.056
**5-6**	2.201	2.625	2.807	2.815	2.683	2.910	3.555	2.749	3.028	5.078	2.172	2.166	2.183	2.212	2.214	2.101
**5-7**	0.110	0.283	0.383	1.580	0.195	0.246	1.463	0.155	0.173	0.328	0.112	0.112	0.104	0.142	0.151	0.144
**5-8**	0.056	0.147	0.204	1.338	0.069	0.077	0.807	0.073	0.077	0.080	0.068	0.071	0.057	0.085	0.094	0.106
**6-7**	2.193	2.617	2.798	2.807	2.675	2.902	3.548	2.228	2.226	1.846	2.181	2.175	2.187	2.167	2.156	2.106
**6-8**	0.110	0.283	0.382	1.579	0.195	0.246	1.462	0.121	0.122	0.097	0.112	0.112	0.106	0.105	0.103	0.084
**7-8**	2.198	2.622	2.803	2.812	2.684	2.910	3.555	2.174	2.171	2.161	2.170	2.164	2.162	2.152	2.141	2.089

**Table 3:** The comparison of image reconstruction results based on the 3D simulation.

Flow Pattern	Algorithm	Relative Image Error	Correlation Coefficient	Estimated Phase Ratio	Phase Ratio Error
50% annular	LBP	37.30%	0.8760	47.25%	−2.75%
	Landweber	22.40%	0.9518	46.21%	−3.79%
	TV	22.45%	0.9516	46.19%	−3.81%
	Autoencoder	10.88%	0.9881	50.88%	0.88%
19.58% stratified	LBP	40.19%	0.9095	17.67%	−1.91%
	Landweber	33.04%	0.9346	17.08%	−2.50%
	TV	32.99%	0.9355	16.94%	−2.64%
	Autoencoder	4.23%	0.9989	19.57%	−0.01%
13.88% single bar	LBP	84.51%	0.6514	7.29%	−6.59%
	Landweber	53.64%	0.9134	7.30%	−6.58%
	TV	37.20%	0.9322	10.54%	−3.34%
	Autoencoder	29.25%	0.9530	15.31%	1.44%
27.76% two-bar	LBP	72.04%	0.7109	17.02%	−10.74%
	Landweber	52.02%	0.8615	17.52%	−10.24%
	TV	47.13%	0.8655	20.27%	−7.49%
	Autoencoder	30.64%	0.9352	24.84%	−2.91%

**Table 4:** The phantom parameters of the static experiment part of the benchmark dataset.

Flow Pattern	Parameters				Phase Ratio
Annular	**T**				
	0.05				11.10%
	0.14				26.53%
	0.29				48.95%
Stratified	**H**				
	0.25				19.58%
	0.50				50%
	0.75				80.42%
Single Bar	**C(x,y)**		**R**		
	(0,0)		0.29		7.93%
	(0,0)		0.43		18.31%
	(0.46,0)		0.50		25%
Two-bar	**C1(x,y)**	**C2(x,y)**	**R1**	**R2**	
			0.29	0.43	26.52%
	(−0.46,0)	(0.46,0)	0.29	0.50	33.15%
			0.43	0.50	43.18%

**Table 5:** The capacitance data related to the examples in Figure 11.

Electrode Pair	Empty	Full	48.95% Annular	19.58% Stratified	18.31% Single Bar	26.52% Two-Bar
**1-2**	2.557	2.930	3.153	3.341	2.518	2.516
**1-3**	0.114	0.322	0.231	0.169	0.119	0.157
**1-4**	0.061	0.189	0.084	0.083	0.083	0.088
**1-5**	0.049	0.147	0.057	0.066	0.074	0.116
**1-6**	0.058	0.174	0.075	0.079	0.079	0.084
**1-7**	0.116	0.338	0.228	0.176	0.121	0.129
**1-8**	2.720	3.188	3.353	3.424	2.689	2.631
**2-3**	2.289	2.648	2.807	2.338	2.255	2.245
**2-4**	0.118	0.340	0.240	0.131	0.123	0.114
**2-5**	0.058	0.169	0.075	0.065	0.078	0.103
**2-6**	0.048	0.140	0.055	0.054	0.071	0.062
**2-7**	0.058	0.174	0.076	0.071	0.079	0.065
**2-8**	0.116	0.332	0.232	0.325	0.121	0.143
**3-4**	2.684	3.131	3.250	2.746	2.655	2.611
**3-5**	0.112	0.316	0.215	0.104	0.118	0.175
**3-6**	0.057	0.168	0.073	0.049	0.078	0.079
**3-7**	0.048	0.146	0.056	0.037	0.073	0.050
**3-8**	0.059	0.177	0.079	0.069	0.079	0.068
**4-5**	2.660	3.194	3.333	2.716	2.624	2.700
**4-6**	0.119	0.352	0.237	0.114	0.125	0.204
**4-7**	0.062	0.194	0.083	0.054	0.084	0.087
**4-8**	0.052	0.162	0.062	0.058	0.078	0.073
**5-6**	2.631	3.132	3.242	2.652	2.601	2.668
**5-7**	0.118	0.350	0.236	0.110	0.123	0.180
**5-8**	0.060	0.183	0.078	0.066	0.081	0.111
**6-7**	2.662	3.194	3.391	2.774	2.625	2.618
**6-8**	0.116	0.340	0.227	0.126	0.121	0.114
**7-8**	2.588	3.128	3.320	2.586	2.552	2.603

**Table 6:** The comparison of the image reconstruction results based on the static experiment.

Flow Pattern	Algorithm	Relative Image Error	Correlation Coefficient	Estimated Phase Ratio	Phase Ratio Error
48.95% annular	LBP	34.07%	0.8954	50.51%	1.56%
	Landweber	16.41%	0.9733	49.42%	0.47%
	TV	16.30%	0.9737	49.43%	0.48%
	Autoencoder	26.41%	0.9338	45.01%	−3.94%
19.58% stratified	LBP	36.82%	0.9193	19.48%	−0.10%
	Landweber	33.67%	0.9279	19.51%	−0.07%
	TV	33.94%	0.9266	19.19%	−0.39%
	Autoencoder	33.16%	0.9360	14.02%	−5.56%
18.31% single bar	LBP	80.69%	0.7151	9.26%	−9.05%
	Landweber	54.18%	0.9118	9.47%	−8.84%
	TV	33.49%	0.9427	14.89%	−3.41%
	Autoencoder	30.14%	0.9438	25.99%	7.68%
26.52% two-bar	LBP	72.34%	0.7075	15.96%	−10.56%
	Landweber	54.54%	0.8310	16.70%	−9.82%
	TV	54.71%	0.8305	16.66%	−9.86%
	Autoencoder	39.11%	0.9031	16.52%	−10.00%

**Table 7:** The capacitance dataset of the permittivity distribution information not open to the public (in pF).

Electrode Pair	Experimental Phantom No. 1	Experimental Phantom No. 2	Experimental Phantom No. 3
**1-2**	2.876	2.873	3.052
**1-3**	0.185	0.126	0.187
**1-4**	0.105	0.060	0.124
**1-5**	0.080	0.053	0.105
**1-6**	0.088	0.083	0.113
**1-7**	0.161	0.128	0.195
**1-8**	3.069	3.026	3.336
**2-3**	2.589	2.589	2.267
**2-4**	0.153	0.132	0.146
**2-5**	0.081	0.060	0.094
**2-6**	0.075	0.068	0.087
**2-7**	0.099	0.059	0.102
**2-8**	0.196	0.129	0.332
**3-4**	3.030	2.958	2.688
**3-5**	0.121	0.123	0.105
**3-6**	0.063	0.083	0.064
**3-7**	0.065	0.051	0.056
**3-8**	0.097	0.057	0.098
**4-5**	3.057	2.971	2.663
**4-6**	0.118	0.179	0.113
**4-7**	0.066	0.075	0.069
**4-8**	0.072	0.050	0.091
**5-6**	2.995	2.933	2.561
**5-7**	0.107	0.188	0.108
**5-8**	0.070	0.074	0.093
**6-7**	3.105	2.985	2.700
**6-8**	0.129	0.166	0.136
**7-8**	2.959	2.927	2.584

**Table 8:** The GVF and corresponding flow rate of the dynamic experiment samples.

GVF	Gas Flow Rate (m^3^/h)	Oil Flow Rate (m^3^/h)
23.71%	5.78	18.61
44.24%	14.33	18.06
62.09%	28.01	17.10

**Table 9:** The normalized capacitance data sequence with 62.09% GVF.

Electrode Pair	Normalized Capacitance Data Sequence in 10 s									
	t = 1 s	t = 2 s	t = 3 s	t = 4 s	t = 5 s	t = 6 s	t = 7 s	t = 8 s	t = 9 s	t = 10 s
**1-2**	2.191	2.615	2.796	2.807	2.681	2.907	3.552	2.182	2.178	2.176
**1-3**	0.110	0.282	0.382	1.579	0.195	0.246	1.463	0.103	0.101	0.096
**1-4**	0.056	0.147	0.203	1.337	0.069	0.077	0.806	0.062	0.064	0.061
**1-5**	0.046	0.122	0.170	1.273	0.052	0.055	0.627	0.060	0.063	0.063
**1-6**	0.056	0.147	0.203	1.338	0.069	0.077	0.807	0.062	0.064	0.061
**1-7**	0.110	0.283	0.383	1.579	0.195	0.247	1.463	0.103	0.101	0.097
**1-8**	2.199	2.623	2.804	2.812	2.686	2.912	3.556	2.187	2.187	2.184
**2-3**	2.193	2.617	2.799	2.808	2.681	2.907	3.552	2.182	2.179	2.169
**2-4**	0.110	0.282	0.382	1.579	0.195	0.246	1.462	0.122	0.124	0.097
**2-5**	0.056	0.147	0.203	1.338	0.069	0.077	0.807	0.073	0.077	0.081
**2-6**	0.046	0.122	0.170	1.274	0.052	0.055	0.627	0.052	0.054	0.068
**2-7**	0.056	0.147	0.204	1.339	0.069	0.077	0.807	0.048	0.047	0.044
**2-8**	0.110	0.283	0.383	1.580	0.195	0.246	1.462	0.106	0.105	0.102
**3-4**	2.196	2.620	2.801	2.810	2.681	2.908	3.552	2.231	2.228	1.765
**3-5**	0.110	0.283	0.383	1.579	0.195	0.246	1.462	0.158	0.177	0.366
**3-6**	0.056	0.147	0.203	1.338	0.069	0.077	0.807	0.065	0.071	0.258
**3-7**	0.046	0.122	0.170	1.274	0.052	0.055	0.628	0.036	0.034	0.041
**3-8**	0.056	0.147	0.203	1.338	0.069	0.077	0.807	0.048	0.047	0.044
**4-5**	2.196	2.620	2.801	2.809	2.680	2.906	3.550	2.742	3.016	4.974
**4-6**	0.110	0.283	0.383	1.579	0.195	0.246	1.462	0.268	0.370	2.984
**4-7**	0.056	0.147	0.204	1.338	0.069	0.077	0.807	0.065	0.070	0.227
**4-8**	0.046	0.122	0.170	1.274	0.052	0.055	0.627	0.052	0.053	0.066
**5-6**	2.201	2.625	2.807	2.815	2.683	2.910	3.555	2.749	3.028	5.078
**5-7**	0.110	0.283	0.383	1.580	0.195	0.246	1.463	0.155	0.173	0.328
**5-8**	0.056	0.147	0.204	1.338	0.069	0.077	0.807	0.073	0.077	0.080
**6-7**	2.193	2.617	2.798	2.807	2.675	2.902	3.548	2.228	2.226	1.846
**6-8**	0.110	0.283	0.382	1.579	0.195	0.246	1.462	0.121	0.122	0.097
**7-8**	2.198	2.622	2.803	2.812	2.684	2.910	3.555	2.174	2.171	2.161
